# STING agonist, SMA-2, inhibits clear cell renal cell carcinoma through improving tumor microenvironment

**DOI:** 10.1007/s11010-024-04970-w

**Published:** 2024-04-09

**Authors:** Wei Wang, Fengqing Zhang, Yan Hu, Guangming Liu

**Affiliations:** 1 Department of Urology, Tianjin First Central Hospital, NO.24 Fukang Road, Nankai District, Tianjin, 300192 People’s Republic of China; 2 Department of Surgery, Tianjin Prevention and Treatment Center for Occupational Diseases, Hedong District, Tianjin, 300011 People’s Republic of China; 3 Clinical Lab, Tianjin Rehabilitation Recuperation Center, Nankai District, Tianjin, 300381 People’s Republic of China

**Keywords:** Tumor progression, STING agonist, Tumor microenvironment, Cytokine, Immune therapy

## Abstract

Clear cell renal cell carcinoma (ccRCC) is the most prevalent and lethal subtype of kidney cancer, patients with ccRCC usually have very poor prognosis and short survival. Therefore, it is urgent to develop more effective therapeutics or medications to suppress ccRCC progression. Here, we demonstrated that STING agonist, MSA-2 significantly inhibits tumor progress and prolongs the survival of ccRCC mice by promoting cytokines secretion. Moreover, MSA-2 triggered the trafficking and infiltration of CD8^+^ T cells, supported by the generation of a chemokine milieu that promoted recruitment and modulation of the immunosuppressive TME in ccRCC. These findings suggest that MSA-2 potentially serves an effective and preferable adjuvant immunotherapy of ccRCC.

## Introduction

Renal cell carcinoma (RCC) ranks as the most common type of urogenital malignancies, which accounts for over 80% of renal malignancies and approximately 2.4% of all malignancies in the world [1, 2]. Clear cell RCC (ccRCC) is the most prevalent and lethal subtype of RCC, representing 75% of RCC [3]. Although radical or partial nephrectomy has significantly limited morbidity, these patients’ prognosis is still poor [4]. Therefore, there is an unmet need to develop more effective therapeutics.

Tumor microenvironment (TME) is composed of various cells including various immune cells and stromal cells [5]. Accumulating data shows that tumor infiltrating lymphocytes, especially cytotoxic T lymphocytes (CTLs) and secreted cytokine are crucial for repressing tumor progression and improving patients’ survival. However, to get rid of immunosurveillance, cancer cells impede both CTLs infiltration and functionality via producing different kinds of factors [6, 7]. In order to inhibit tumor progression and extend patients’ survival, therefore, the medication which could potent CTLs activity and improve TME should be discovered and developed as quick as possible.

The cGAS-STING pathway as an innate immune sensor plays a crucial role in triggering anti-tumor immunity [8–10]. Activation cGAS-STING pathway could induce chemokines, pro-inflammatory cytokines such as interferon (IFN) secretion and arouse T cells infiltration regulating host immunity [11, 12]. MSA-2 emerges as an oral non-nucleotide STING agonist which could stimulate IFN-β secretion in tumors and induce anti-tumor immunity [13]. MSA-2 is suitable for systemic administration in cancer immunotherapy, providing a promising adjuvant for promoting anti-tumor immunity [14].

Although having shown promising effect in colorectal cancer therapy [15], whether it would have similar efficacy in other tumor type, such as ccRCC, remains to be explored. Herein, we observed that MSA-2 elicits robust anti-tumor efficacy and prolongs survival of mice with ccRCC under different drug-given pathways including intratumoral (IT) injection, subcutaneous (SC) injection and oral (PO) and revealed. Importantly, our data prove that MSA-2 inhibits ccRCC progression depending on intact immune system and CD8^+^ T cells presence. Mechanistically, we found that MSA-2 stimulated a variety of chemokines and cytokines production which not only promotes CD8^+^ T cells infiltration but improved their activity in tumor microenvironment. Therefore, these pre-clinical data collectively demonstrated that MSA-2 would be a very promising candidate in ccRCC therapy and warrant to obtain more attentions in the future study.

## Materials and methods

### Study approvals

All experimental procedures were approved and in accordance with China’s National Code of Animal Care for Scientific Experimentation. The experiments were also assessed by the Animal Experimentation Ethics Committee of Nankai University, and the assigned approval number is 2023-SYDWLL0006603.

### Cell culture

RENCA cells were purchased from the American Type Culture Collection (ATCC) and maintained in DMEM containing 10% fetal bovine serum, 100 units/mL penicillin, and 100 ug/mL streptomycin at 37 °C with 5% CO_2_ in an incubator.

### Animal studies

Blbc/c mice and nude mice were purchased from Institute of Radiological Medicine, Chinese Academy of Medical Sciences and housed in a specific pathogen-free (SPF) condition (12 h light/12 h dark cycle, temperature 20–25 °C) and had free access to water and chow. For the syngeneic subcutaneous tumor model, all age-matched Blbc/c mice (6–8-week-old) were shaved at the right flank. RENCA(5X10^5^) tumor cells were injected into the shaved, right flank of indicated mice. After the tumor reached around 100 mm^3^, mice were randomly separated into groups with different treatments. MSA-2 or vehicle were administrated via I.T. injection, S.C. injection, or P.O. gavage every three days for total 3 times. Tumor volume was measured by caliper and calculated using the formula: width^2^ × length/2. Mice were euthanized when > 30% of body weight was lost or when tumor volume approached ~ 1000–1500 mm^3^, or when tumors became ulcerated.

### CD4^+^ or CD8^+^-T cell depletion

Blbc/c mice administrated with IgG, anti-CD4 Ab (clone GK1.5, Sungene, China) or anti-CD8 Ab (clone 2.43, Sungene, China) antibodies intraperitoneally at the dose of 200 μg/mouse/week for 2 weeks to deplete CD8^+^ and CD4^+^ T cells. Day 0, RENCA cells (5 × 10^5^) were subcutaneously injected into the right flank of these mic and these tumor-bearing mice were treated with vehicle or SMA-2 (45 mg/kg, every 3 days for total 3 times). Tumors volume and body weight were measured for every other day with caliper and a weigh scale, respectively. Mice were euthanized when tumor reached 1000 mm^3^ or ulceration occurred.

### ELISA assay

Serum collection: Blood was harvested from mouse at 0, 4, 8, 12 and 16 h after treated with vehicle or SMA-2 followed by centrifugation (3000 rpm for 30 min). Then the upper serum was collected carefully and subjected to IFN-β, IL-6 and TNF-α test using Quantikine® ELISA kit (R&D Systems, MIFNB0, M6000B and MTA00B) according to the manufacture’s protocol.

For tumor homogenate: The tumor tissue was isolated, dissected, ground, and suspended in 2 mL cold PBS followed by centrifugation (2000 rpm for 5 min). After that, supernatant was collected and subjected to IFN-β, IL-6 and TNF-α test using the same kit described above.

### Flow cytometry analysis

For immunoprofiling of solid tumors, tumor tissue was dissected and digested with 1 mg/mL Collagenase D (Roche) plus with 100 mg/mL DNase I (Roche) in RPMI medium with 2% FBS for 1 h with continuous agitation at 37 °C. Digestion mixture was passed through 100 mm cell strainer to prepare single cell suspension and washed with PBS supplemented with 2 mM EDTA and 1% FBS. Anti-mouse CD16/32 antibodies were used to block single cells for 10 min prior to surface molecules staining. Cell surface staining was performed by incubating single cell suspensions with rat anti-mouse CD45 (Biolegend, #103149), rat anti-mouse CD3 (Biolegend, #100206), CD8 (Biolegend, #100712), CD4 (Biolegend, #100406) and NK1.1 (Biolegend, # 156514) fluorophore-conjugated antibodies on ice for 30 min. Flow cytometric analysis was performed on a LSR Fortessa™ flow cytometer (BD Biosciences) and analyzed using FlowJo software (TreeStar).

### Statistical analysis

All experiments described here are the representative of at least three independent experiments (*n* = 5 mice for each group). For in vitro experiments, cells or tissues from each of these animals were processed (at least) in biological triplicates. All data were shown as average ± S.E.M. Statistical analysis between two groups was performed with 2-tailed Student t test and multiple comparisons was conducted using One-way ANOVA or two-way ANOVA analysis with Tukey’s multiplecomparison. Tumor growth was analyzed with Repeated-measure two-way ANOVA (mixed-model) with Tukey’s multiple-comparison. The Kaplan–Meier curves were used to analyze the survival data, and Cox regression was used to compute hazard ratio. All graphs and statistical analysis were performed using GraphPad Prism 9 and the results of statistical analyses for each experiment are clearly indicated in the respective figures and in Figure Legends. *p* values < 0.05 were considered significant.

## Results

### MSA-2 robustly inhibited ccRCC tumor growth and prolonged mice survival

**Fig. 1:**
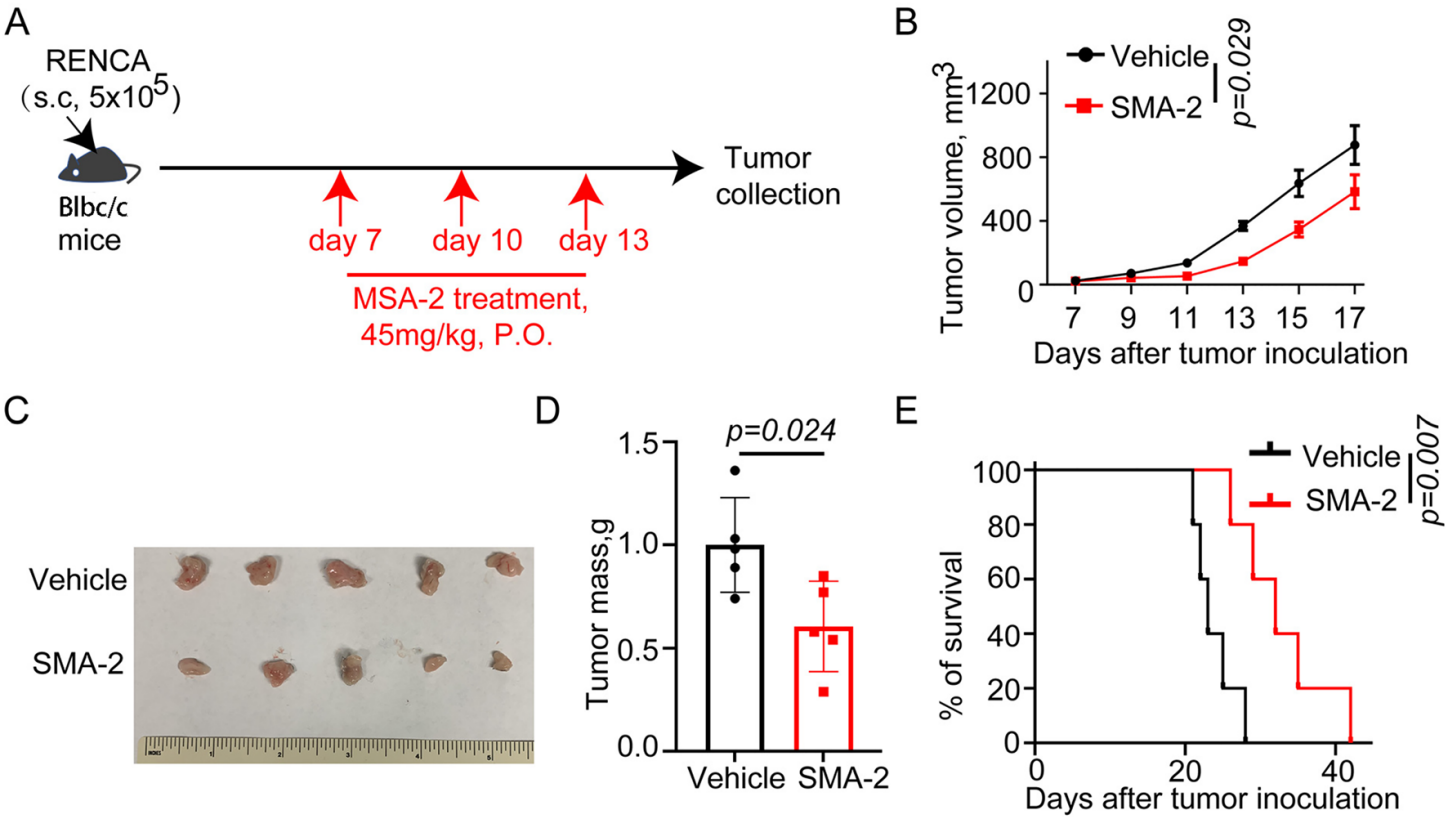
MSA-2 inhibits ccRCC growth and prolongs survival of tumor-bearing mice. **A** A schematic of experiment demonstrating the role of MSA-2 on tumor growth. **B** Analysis of subcutaneous tumor growth in mice treated with Vehicle or MSA-2(45 mg/kg) (*n* = 5). **C** Representative tumor image from panel A (*n* = 5). **D** Analysis of tumor weight from panel A (*n* = 5). **E** Survival analysis of mice treated with Vehicle or MSA-2 (*n* = 5). Data are presented as mean ± SEM. Statistical analysis was performed using 2-tailed Students’ *t* test (**D**) or 2-way ANOVA with Sidak’s multiple-comparison test (**B**) or log-rank (Mantel-Cox) test (**E**)

To investigate the anti-tumor efficacy of MSA-2 in vivo, we established subcutaneous RENCA tumor model and treated these tumor-bearing mice with vehicle or MSA-2 (Fig. 1A). Our data showed that, compared to vehicle treatment, MSA-2 treatment modest but significantly suppressed ccRCC growth (Fig. 1B) and reduced tumor volume and mass (Fig. 1C, D). Consistent with MSA-2-induced anti-tumor efficacy, MSA-2 also notably prolonged survival of tumor-bearing mice (Fig. 1E). Collectively, our data indicate that MSA-2 possesses a strong anti-tumor function in vivo.

### Oral injection is the most effective pathway for MSA-2-based anti-tumor efficacy in different dose

**Fig. 2:**
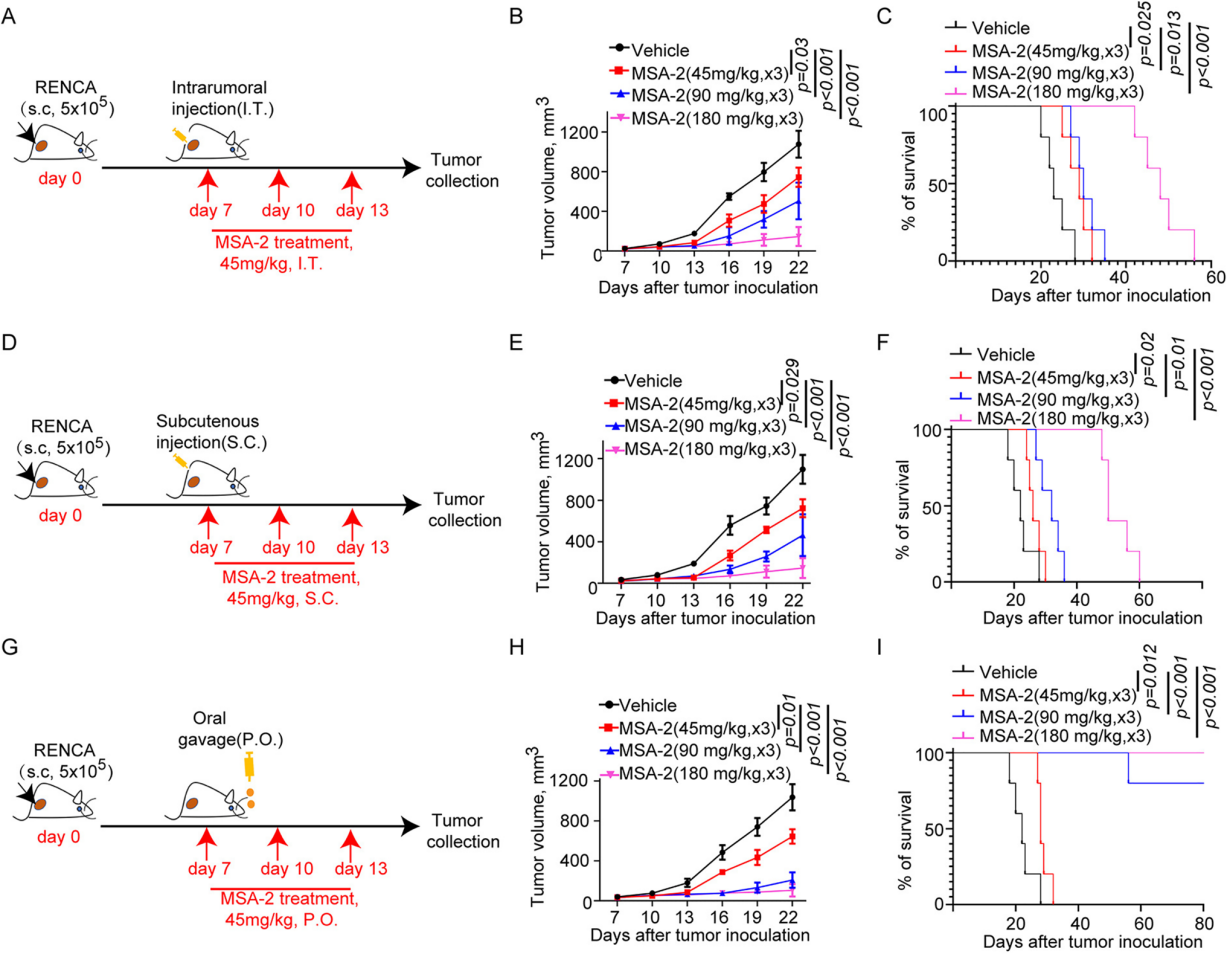
Oral injection is the most effective pathway for MSA-2-based anti-tumor efficacy under different doses. **A** A Schematic of treatment with different dose MSA-2 through intratumoral injection. **B** Analysis of tumor growth in mice treated with vehicle or different dose of MSA-2 through I.T injection (*n* = 5). **C** Survival analysis of tumor-bearing mice treated with vehicle or different dose of MSA-2 through S.C. injection (*n* = 5). **D** Schematic of treatment with different dose MSA-2 through subcutaneous injection. **E** Analysis of tumor growth in mice treated with vehicle or different dose of MSA-2 through P.O injection (*n* = 5). **F** Survival analysis of tumor-bearing mice treated with vehicle or different dose of MSA-2 through S.C injection through I.T. injection (*n* = 5). **G** Schematic of treatment with different dose MSA-2 through oral gavage. **H** Analysis of survival of mice treated with Vehicle or different dose of MSA-2 through S.C injection (*n* = 5). **I** Survival analysis of tumor-bearing mice treated with vehicle or different dose of MSA-2 through S.C injection through P.O. injection (*n* = 5). Data are presented as mean ± SEM. Statistical analysis was performed using 2-way ANOVA with Sidak’s multiple-comparison test (**B**, **E** and **H**) or log-rank (Mantel-Cox) test (**C**, **F** and **I**)

MSA-2-induced anti-tumor efficacy may be associated with drug-given pathway and dose. Next, we evaluated the anti-tumor potential of MSA-2 at different doses through different drug-given pathway including intratumoral (IT, Fig. 2A), subcutaneous (S.C., Fig. 2D), or oral (P.O., Fig. 2G) in the syngeneic RECAN mouse tumor models. We found that MSA-2 displayed dose-dependent antitumor efficacy when administered by IT, SC, or PO routes manifested by both tumor growth (Fig. 2B, E and H) and survival curves (Fig. 2C, F and I). Intriguingly, the tumors treated with MSA-2 at 90 mg/kg for 3 times by PO administration were dramatically regressed and all these mice’ survive was significantly extended compared to other administration pathway in the same treatment regime (Fig. 2G–I). Thus, our data showed that MSA-2 elicited tumor suppression depends on dose and P.O. administration is the most effective manner in preventing tumor progression or extend survival of tumor-bearing mice.

### MSA-2-induced anti-tumor efficacy depending on immune system

**Fig. 3:**
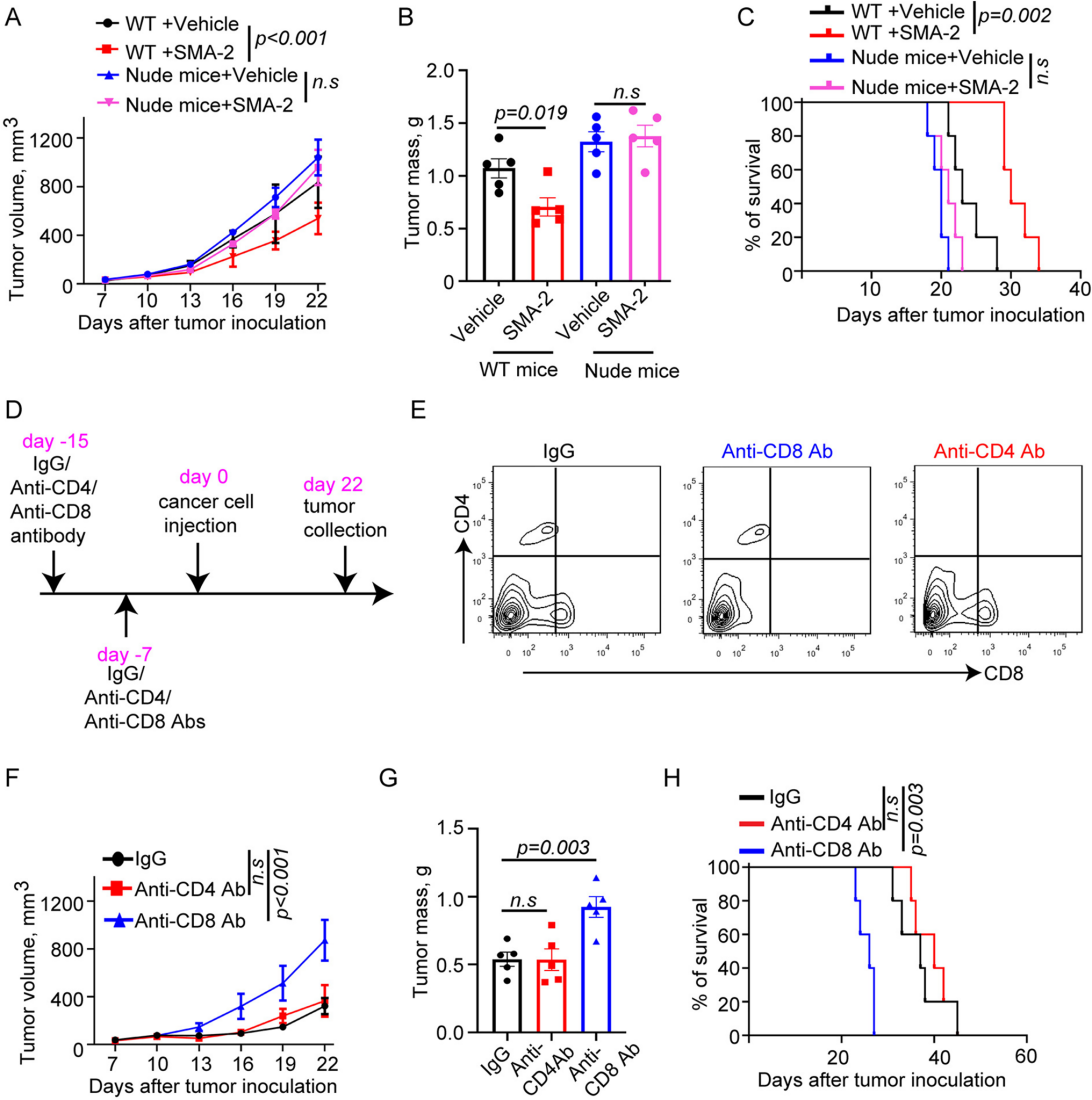
MSA-2-induced anti-tumor efficacy depends on immune system. **A** Analysis of tumor growth in nude or WT mice treated with vehicle or MSA-2(45 mg/kg, P.O.) (*n* = 5). **B** Analysis of tumor weight from panel A (*n* = 5). **C** Survival analysis of WT or Nude mice treated with vehicle or MSA-2(45 mg/kg, P.O.) (*n* = 5). **D** Schematic of experiment assay the role of CD8 + T cells on MSA-2-induced anti-tumor efficacy. **E** Flow cytometry analysis of percentage of CD4 + or CD8 + T cells in spleen from mice treated with anti-CD4 or anti-CD8 Ab. **F** Analysis of tumor growth in mice treated with MSA-2 combining anti-CD4 or anti-CD8 antibody (*n* = 5). **G** Analysis of tumor weight from experiment E (*n* = 5). **H** Survival analysis of mice treated with MSA-2 combining anti-CD4(200 μg/kg) or anti-CD8(200 μg/kg) antibody (*n* = 5). Data are presented as mean ± SEM. Statistical analysis was performed using 2-tailed Students’ *t* test (**B** and **G**) or 2-way ANOVA with Sidak’s multiple-comparison test (**A** and **F**) or log-rank (Mantel-Cox) test (**C** and **H**)

Given MSA-2 triggered strong immune response, we next want to know if MSA-2-induced anti-tumor efficacy is immune system dependent. To this end, we established RENCA cells tumor model using nude mice which lacks normal thymus and normal T development. Consistent with previous data, MSA-2 significantly reduced tumor growth and mass in WT mice, however, this efficacy was largely reversed in nude mice model (Fig. 3A, B). Accordingly, MSA-2 could notably extend lifespan of WT mice but not nude mice (Fig. 3C). This data indicated that immune system is required for MSA-2-induced tumor prevention.

To further verify the cell causing the tumor suppression, CD4^+^ or CD8^+^ T cells were depleted using anti-CD4 or anti-CD8 neutralization antibody (Fig. 3D, E). Interestingly, whereas depletion CD4^+^ T cells did not impact the anti-tumor efficacy, CD8^+^ T cells depletion dramatically abolished MSA-2 caused tumor inhibition, accelerated tumor growth and increased tumor mass (Fig. 3F, G). In addition, we observed that MSA-2 could not extend mice survival of tumor-bearing mice without CD8^+^ T cells (Fig. 3H). Collectively, these data suggested that MSA-2-based anti-tumor function relies on the presence of immune system and CD8^+^ T cells.

### MSA-2 stimulates cytokine production in serum and tumor tissue

**Fig. 4:**
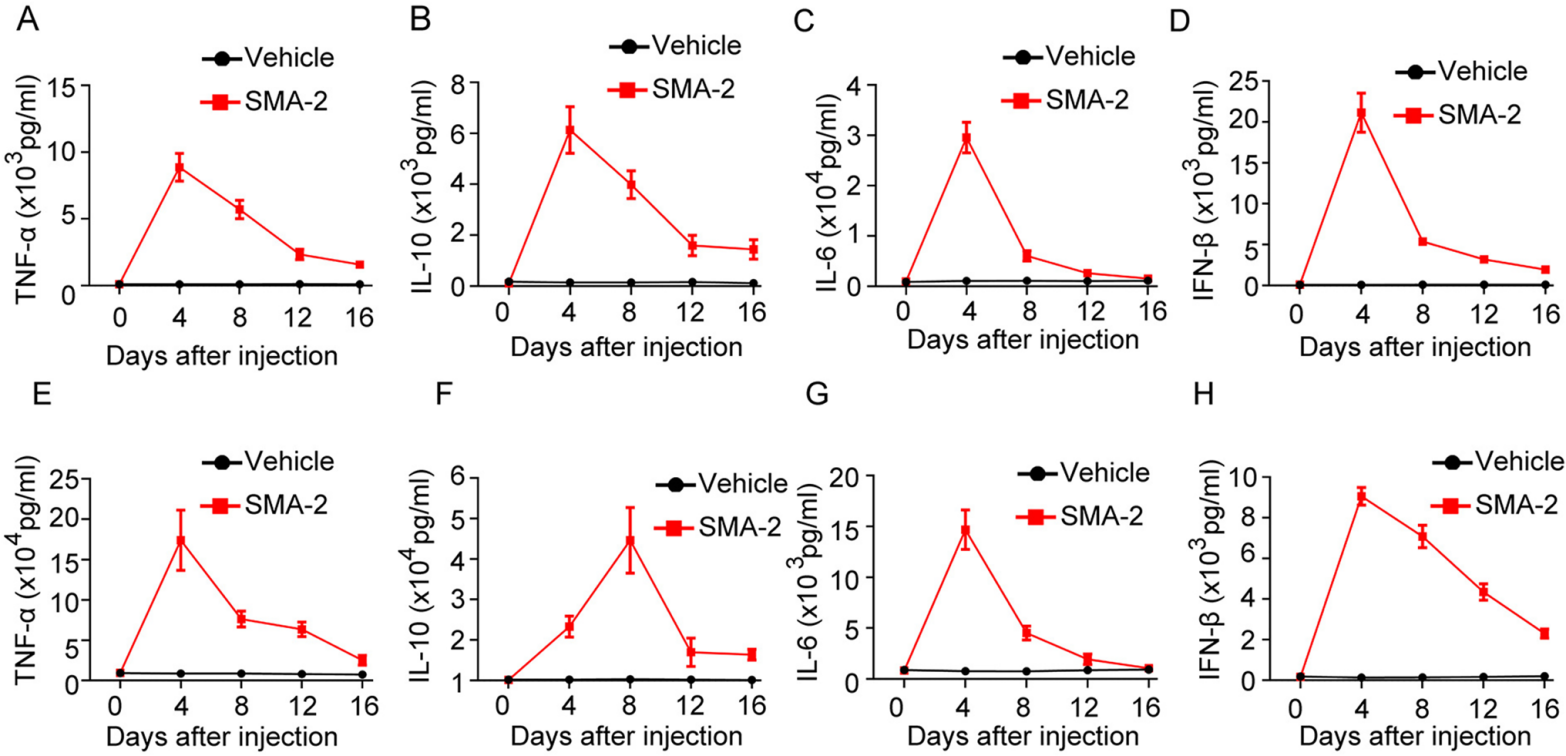
MSA-2-induced multiple cytokines production in vivo. **A** Test of TNF-α level in plasma at different time points after treated with Vehicle or MSA-2. **B** Test of IL-10 level in plasma at different time points after treated with Vehicle or MSA-2. **C** Test of IL-6 level in plasma at different time points after treated with Vehicle or MSA-2. **D** Test of IFN-β level in plasma at different time points after treated with Vehicle or MSA-2. **E** Analysis of TNF-α level in tumor at different time points after treated with Vehicle or MSA-2. **F** Analysis of IL-10 level in tumor at different time points after treated with Vehicle or MSA-2. **G** Analysis of IL-6 level in tumor at different time points after treated with Vehicle or MSA-2. **H** Analysis of IFN-β level in tumor at different time points after treated with Vehicle or MSA-2. Data are presented as mean ± SEM and *n* = 5 for each group

As STING agonist can activate innate immune pathway and incased multiple cytokines secretion [14], we next determined the level of multiple cytokines in peripheral and tumor microenvironment. Through different time points analysis, we found that MSA-2 significantly stimulated TNF-α, IL-10, IL-6 and INF-β expression in both plasma (Fig. 4A–D) and tumor (Fig. 4E–H) and this expression reached the highest level around 4–6 days after MSA-2 injection and down to normal spectrum after 16 days. This data indicates that MSA-2 could stimulate immune cells and promote multiple cytokines secretion in both peripheral and tumor microenvironment.

### MSA-2 improves CD8^+^ T cells infiltration and activity in tumor microenvironment

**Fig. 5:**
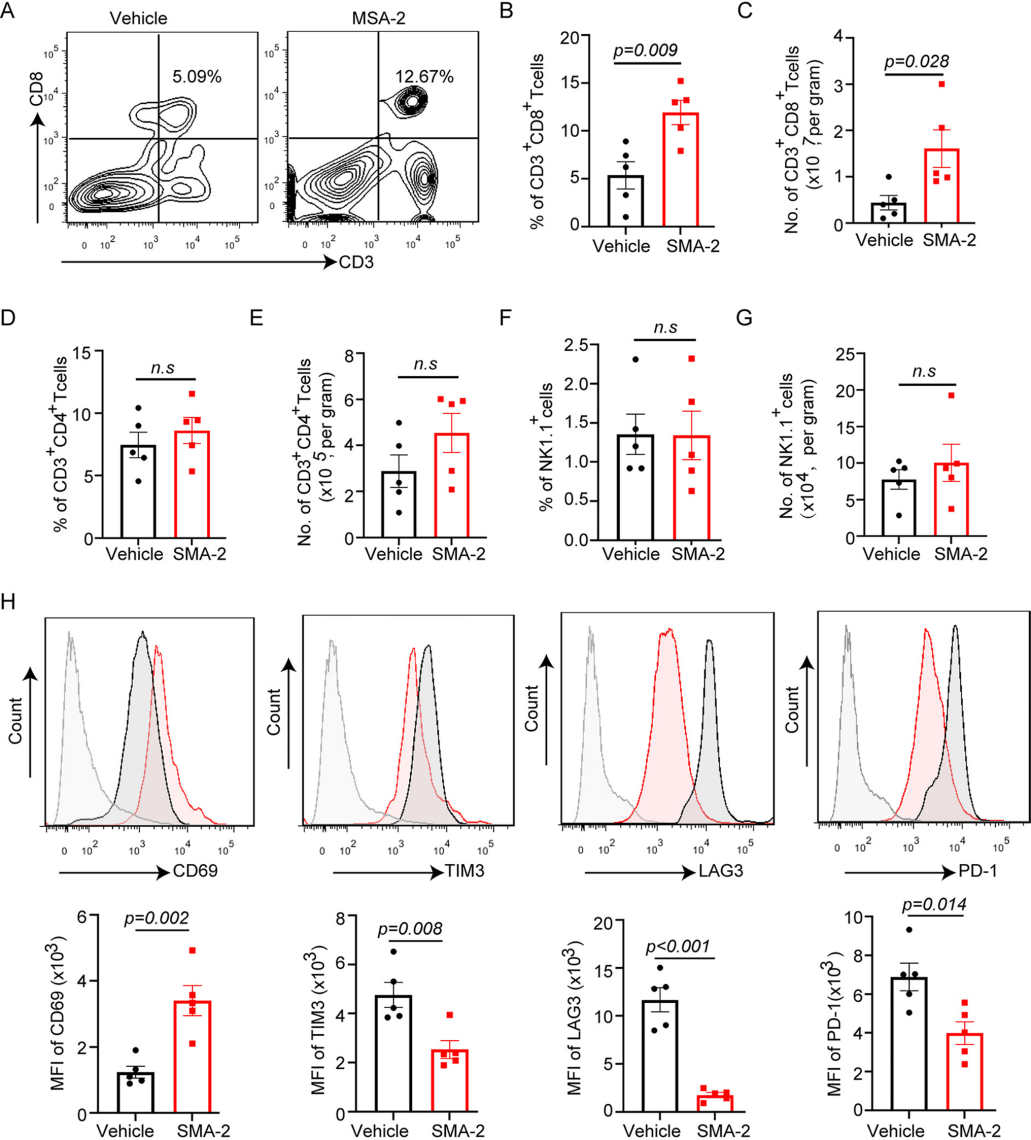
MSA-2 improves CD8 + T cells in tumor microenvironment. **A** Flow cytometry analysis of percentage of CD3 + CD8 + T cells in tumor from mice treated with vehicle or MSA-2(45 mg/kg, P.O.) (*n* = 5). **B** Quantification of percentage of CD3 + CD8 + T cells in tumor from panel A (*n* = 5). **C** Quantification of absolute cell number of CD3^+^CD8^+^ T cells in tumor from mice treated as panel A (*n* = 5). **D** Flow cytometry analysis of percentage of CD3^+^CD4^+^ T cells in tumor from mice treated with vehicle or MSA-2(45 mg/kg, P.O.) (*n* = 5). **E** Flow cytometry analysis of cell number of CD3 + CD4 + T cells in tumor from mice treated as panel C (*n* = 5). **F** Flow cytometry analysis of percentage of NK cells in tumor from mice treated with vehicle or MSA-2(45 mg/kg, P.O.) (*n* = 5). **G** Flow cytometry analysis of cell number of NK cells in tumor from mice treated as panel E (*n* = 5). Data are presented as mean ± SEM. Statistical analysis was performed using 2-tailed Students’ *t* test

Given the amount and activity of CD8^+^ T cells in tumor microenvironment are critical for tumor progression [16] and MSA-2 elicited tumor defeat relies on the presence of CD8^+^ T cells (Fig. 3E), we further decoded the mechanism by which MSA-2 repressed tumor progression through analyzing the immunoprofile of tumor microenvironment. MSA-2 treatment significantly increased the percentage and number of CD8^+^ T cells (Fig. 5A–C) whereas did not impact either percentage or number of CD4^+^ T cells and NK cells (Fig. 5D–G). Importantly, further analysis of CD8^+^ T cells status shows that MSA-2 treatment notably increased CD69 expression while suppressing the level of exhaustion markers TIM3, LAG3 and PD-1 (Fig. 5H). These results indicate that MSA-2 inhibited tumor progression through increasing CD8^+^ T cells infiltration as well as their activity.

## Discussion

In spite of current improvement of clinical outcome offered by antibody-based targeted therapies, such as VEGF targeted therapies, ccRCC still suffers from high cancer-related deaths annually, which requires more powerful strategies to improve patient survival outcomes. With the advent of novel immunotherapy, an adjuvant setting has been guided to provide treatment strategies for ccRCC patients. Cytokine-based immunotherapy agents such IFN-α, emerges as a standard care for the advanced RCC treatment; however, several studies showed that these cytokine stimulators either or in combination, failed to improve the disease-free survival or overall survival. cGAS-STING pathway has been recognized as a promising target for improving immune response and suppressing tumor progression. To date, a variety of STING agonists were designed and developed for cancer therapy, showing great treatment responses in pre-clinical work. Unfortunately, the administration path of conventional STING agonists is typically adopted as intratumoral injection, highly limiting their application in the clinic. MSA-2 is an oral non-nucleotide STING agonist, which sharply overcomes the administration shortcoming of conventional STING agonists and has been considered as a milestone in cancer immunotherapy due to promising systemic administration. In this current study, we found that oral administration of MSA-2 effectively inhibited the tumor growth and improved the survival of the tumor-bearing mice in syngeneic ccRCC models.

The significant toxicity and the low efficacy methodological treatment of pretargeted drugs also failed to provide clinical benefit in ccRCC cancer immunotherapy. Oral administration is considered a convenient low-cost delivery route. Oral mucosal drug directly enters the systemic circulation by through the gastrointestinal tract and metabolic in liver. Drugs for oral delivery are limited to develop owing to its complicated process and high cost of product. Intratumoral delivery are attractive options to improve the drug bioavailability in situ and anti-tumor efficacy. Subcutaneous administration route is widely used for short and rapid drug delivery with high bioavailability. Several factors affect the drug efficacy: injection methodology, pH of formulation and tissue damage. In this work, we found that oral administration of MSA-2, especially under low does (90 mg/kg), exhibited stronger anti-tumor efficacy compared to intratumoral and subcutaneous administration.

Of note, we found MSA-2 administration significantly increased the secretion of cytokine, including IFN-β, IL-6 and TNF-α, well-known regulators immune response. Therefore, the short persistence of MSA-2 and increased levels of pro-inflammatory cytokines in the tumor-bearing mice further supported the enhanced anti-tumor responses observed in our preliminary results. Moreover, we found the failure of tumor controlling in Nude mice treated with MSA-2, highlighting the critical role of immune system in the anti-tumor activity of MSA-2 in syngeneic ccRCC models. ccRCC has been recognized as an immunogenic tumor as it holds the ability to recruit T cell and tumor-associated macrophages, inducing adaptive anti-tumor immunity. As such, MSA-2 induced the secretion of multiple cytokines, further boosting the immune tumor microenvironment. And in vivo depletion of CD8 + T cells by antibody treatment confirmed that MSA-2 can induced the robust priming, trafficking to and tumor infiltration of CD8 + T cells, underscoring the immune regulation of MSA-2 in the syngeneic ccRCC models.

There are a few limitations for our study. First, we reported the anti-tumor efficacy in RCC murine model by boosting the innate and adaptive immune response. However, the explorations of combination therapy are needed to explore in the adjuvant therapy of advanced ccRCC tumors. Second, our observed enrichment of tumor infiltrated CD8^+^ T cells further highlights the immune activator role of MSA-2. However, the mechanism of MSA-2 in regulating CD8^+^ T cells recruitment, infiltration and elimination is largely unknown and warrants further study. Finally, The TME is more complex and changeable, the role of MSA-2 in regulating the innate and adaptive need to further explore. The functions of DCs, macrophages, NK cells and cancer-associated fibroblasts also warrant systemically investigation.

In summary, our study established STING agonist, MSA-2, greatly enhanced the cytokine secretion, and induced robust anti-tumor immunity for tumor inhibition. Furthermore, MSA-2 facilitated the trafficking and infiltration of CD8+ T cells by fostering the development of a chemokine environment that facilitated the recruitment and modulation of the immunosuppressive tumor microenvironment (TME) in renal cell carcinoma (RCC). This study provides a new approach to clinical management and treatment of RCC or other types of solid tumors.

## Data Availability

All the data were presented in the main Figs. 1–5 and raw data could be obtained from the corresponding author upon reasonable request. All other authors have had full access to all the data in the study and take responsibility for the integrity of the data and the accuracy of the data.

## Declarations

### Research involving human and animal participants

This study does not involve any clinical-related study and no patients were enrolled.
